# Molecular epidemiology of *Clostridioides difficile* colonization in patients with chronic kidney disease in eastern China

**DOI:** 10.1128/spectrum.00418-26

**Published:** 2026-07-17

**Authors:** Weihong Huang, Hangcong Xu, Xinwei Chen, Xiaojun Song, Kunhua Zhu, Dudu Chen, Jianzhong Wang, Yun Luo, Yu Chen, Dazhi Jin

**Affiliations:** 1 Department of Nephrology, The Third People's Hospital of Anji County, Huzhou, Zhejiang, China; 2 School of Public Health, Hangzhou Medical College 117839, Hangzhou, Zhejiang Province, China; 3 Laboratory Medicine Center, Department of Clinical Laboratory, Zhejiang Provincial People’s Hospital, Hangzhou Medical College 117839, Hangzhou, Zhejiang, China; 4 School of Biotechnology and Biomolecular Sciences, University of New South Wales 7800, Sydney, New South Wales, Australia; 5 School of Laboratory Medicine, Hangzhou Medical College 117839, Hangzhou, Zhejiang, China; 6 Zhejiang Key Laboratory of High-level Biosafety and Biomedical Transformation, Hangzhou Medical College 117839, Hangzhou, China; The University of Alabama at Birmingham, Birmingham, Alabama, USA

**Keywords:** *Clostridioides difficile *colonization, molecular characteristics, risk factor, chronic kidney disease

## Abstract

*Clostridioides difficile* colonization (CDC) is considered an important potential reservoir for subsequent infection, yet its role in patients with chronic kidney disease (CKD) remains unclear. A over 2-year cross-sectional study was conducted at one hospital in Zhejiang, China. Fecal specimens collected from outpatients in the nephrology department were subjected to *C. difficile* isolation. Isolates were further characterized through toxin gene detection, multilocus sequence typing, whole-genome sequencing, and antimicrobial susceptibility testing. Demographic data were also collected for statistical analysis. Among the 323 patients with CKD enrolled in this study, approximately 5.0% (16/323) were colonized with *C. difficile*. The 16 *C. difficile* isolates comprised 9 toxin A-negative, toxin B-positive, and binary toxin-negative (A^−^B^+^CDT^−^), 1 A^−^B^+^CDT^+^, 2 A^+^B^+^CDT^−^, and 4 A^−^ B^−^CDT^−^, yielding 8 sequence types. All the isolates were susceptible to vancomycin and metronidazole, and significant associations were found between resistance to erythromycin and tetracycline and toxin genotypes (*P* = 0.009 and 0.002). Multivariable regression analysis revealed that hyperphosphatemia (OR = 1.18; 95% CI, 1.048–1.065) and hemodialysis frequency (OR = 1.10; 95% CI, 1.023–1.467) were independent risk factors for CDC. This study provides the first systematic characterization of the epidemiological profile of CDC in patients with CKD in China; hyperphosphatemia and high-frequency hemodialysis were first identified as independent risk factors for CDC.

**IMPORTANCE:**

*Clostridioides difficile* is one of the main causative agents of antibiotic-associated and hospital-associated diarrhea. This study provides the first systematic molecular epidemiological analysis of *Clostridioides difficile* colonization (CDC) among outpatients with chronic kidney disease (CKD) in eastern China, revealing a colonization rate of 5.0%. Hyperphosphatemia and frequent hemodialysis were established as independent risk factors for CDC. While all isolates retained susceptibility to first-line antimicrobials, they exhibited substantial resistance to fluoroquinolones and clindamycin. These findings address a significant gap in local epidemiological data related to this high-risk cohort and indicate that CKD outpatients may represent an underrecognized potential source for the transmission of antimicrobial-resistant *C. difficile*. Consequently, this study offers an evidence base to strengthen infection prevention and control in nephrology care settings across China. In addition, these preliminary findings also suggest that active surveillance and risk-stratified strategies may be worth considering in other immunocompromised populations.

## INTRODUCTION

*Clostridioides difficile*, a fastidious, gram-positive, spore-forming anaerobic bacterium, is a primary etiological agent of healthcare-associated infectious diarrhea and pseudomembranous colitis (1–4). *C. difficile* infection (CDI) is associated with substantial morbidity and mortality, particularly in immunosuppressed patients. Notably, the highest risk of CDI is observed in hospitalized individuals aged >65 years who have a recent history of antibiotic exposure, as antimicrobial use disrupts the intestinal microbiota barrier, facilitating *C. difficile* colonization and toxin-mediated pathogenesis (5–7).

Currently, immunosuppressed patients are a high-risk group for CDI, with their susceptibility closely linked to underlying diseases and therapeutic medications (8, 9). Previous studies on CDI have primarily focused on kidney transplant recipients, owing to the high recurrence rate and mortality of CDI in this patient cohort (10, 11). Chronic kidney disease (CKD) constitutes a global epidemiological burden, with an estimated prevalence ranging from 8% to 16% (12). A recent meta-analysis concluded that patients with CKD and end-stage renal disease have approximately 1.95- and 2.63-fold higher risk of CDI, respectively, compared to the general population (13). Furthermore, CDI-related mortality is 2.15-fold higher in CKD patients (14). The primary factors contributing to the increased incidence and severity of CDI in CKD patients appear to be impaired immune function, which leads to an inadequate host response to *C. difficile* toxins (13, 15).

In healthy Chinese individuals, the asymptomatic carriage rate of *C. difficile* is approximately 0.7%–7% (16, 17). However, substantially higher rates have been reported in hospitalized cancer patients (20.5%) (18) and patients with hematological malignancies (19), indicating that underlying disease status and healthcare exposure are important determinants of *C. difficile* colonization (CDC). Nevertheless, epidemiological data on CDC in patients with kidney-related diseases remain scarce in China, with research specifically focusing on CKD patients being extremely limited (20, 21). Therefore, this study aims to investigate the prevalence, risk factors, and molecular epidemiological characteristics of CDC in outpatients with CKD in eastern China.

## MATERIALS AND METHODS

### Study participants and data collection

This study was conducted in the Nephrology Department of the Third People’s Hospital of Anji County, Zhejiang Province, from 20 January 2022 to 11 December 2024. Participants were enrolled through random sampling. Stool samples were collected from asymptomatic individuals without symptoms of CDI. CDC was defined as the detection of *C. difficile* in stool samples from asymptomatic individuals (22). Stool specimens were collected using standard sampling tubes and stored at −80°C upon arrival at the laboratory for subsequent analyses. Comprehensive demographic and clinical data were collected for all outpatients, including sex, age, antibiotic use within 90 days, clinical presentation at the time of outpatient visit (such as diagnosis, fever, heart rate, and basic laboratory parameters), underlying comorbidities (including diabetes, chronic obstructive pulmonary disease, heart failure, hypertension, heart disease, and liver disease), and treatment records encompassing dialysis status, dialysis frequency, and antibiotic usage.

### Isolation and culture of *C. difficile*

All stool samples were treated with ethanol and inoculated onto cefoxitin-cycloserine fructose agar plates, which were prepared following previously described protocols (23–25). The inoculated plates were transferred to an anaerobic chamber (bioMérieux, Marcy l’Etoile, France), which was infused with a mixed gas to maintain 0.16% oxygen content, and incubated anaerobically at 37°C for 48 h. Presumptive *C. difficile* colonies were selected based on typical morphology and distinctive odor, then subcultured onto Columbia blood agar for purification (26, 27). Following confirmation of identification, purified colonies were harvested using sterile swabs, suspended in bacterial preservation medium, and stored at −80°C.

### DNA extraction and PCR of *C. difficile* toxin genes

After recovering *C. difficile* isolates on blood agar plates, genomic DNA was extracted using the QIAamp DNA Blood Mini Kit (Valencia, CA, USA) following the manufacturer’s instructions, and the amplification protocols for the housekeeping genes *tpi*, *tcdA*, and *tcdB* were performed as previously described (28–30). The PCR product of the *tpi* gene from *C. difficile* isolates was 230 bp in length, while the *tcdA* gene fragment was 369 bp in toxin A-positive, toxin B-positive (A^+^B^+^) strains and 110 bp in toxin A-negative, toxin B-positive (A^−^B^+^) strains, with the *tcdB* gene fragment consistently 688 bp in both subtypes (31, 32). For binary toxin genes, *cdtA* and *cdtB* amplicons were 353 and 490 bp in a binary toxin-positive strain (CDT^+^), respectively. The standard *C. difficile* strain ATCC BAA-1870 (Manassas, VA, USA) served as the positive control.

### Whole-genome sequencing and multilocus sequence typing

All isolates were sequenced using 150-bp paired-end reads on the Illumina NovaSeq platform. The complete genomes of *C. difficile* were assembled and corrected using Canu v2.2, with the *C. difficile* strain 630 (GenBank accession number CP010905.2) as the reference genome (33). Antimicrobial resistance genes and virulence genes were analyzed via ABRicate (v0.9.8, https://github.com/tseemann/abricate) using ResFinder and VFDB (2024, http://www.mgc.ac.cn/VFs/), respectively, with positive thresholds set at ≥80% sequence identity and coverage, to provide a molecular basis for toxin genotype classification (34). For each identified strain, multilocus sequence typing (MLST) was performed by sequencing seven housekeeping genes (*adk*, *atpA*, *dxr*, *glyA*, *recA*, *sodA*, and *tpi*), in accordance with the procedure previously described by Griffiths et al. (29). The resulting sequences were then submitted to the *C. difficile* MLST database (https://pubmlst.org/cdifficile/) for comparison to derive the corresponding allelic profiles and MLST sequence types.

### Phylogenetic analysis and gene feature visualization

A phylogenetic tree was constructed using the ITOL website (https://itol.embl.de/) based on core genomes. To analyze the genetic characteristics of the *C. difficile* genomes, the presence of virulence genes and antimicrobial resistance genes was integrated into a layered heatmap and visualized alongside the phylogenetic tree to assess associations between genotypes and phenotypic traits.

### Antimicrobial susceptibility testing

The susceptibility of *C. difficile* isolates to 12 antimicrobial agents (vancomycin, metronidazole, rifampin, levofloxacin, ciprofloxacin, moxifloxacin, gatifloxacin, piperacillin, fusidic acid, erythromycin, tetracycline, and clindamycin) was determined. The standard *C. difficile* strain (ATCC 700057) was used as a quality control. Antimicrobial susceptibility testing was performed using the agar dilution method recommended by the Clinical and Laboratory Standards Institute (CLSI) (35). The interpretation of minimum inhibitory concentration (MIC) results was performed according to the criteria summarized in Table 1. Briefly, breakpoints for piperacillin, metronidazole, moxifloxacin, clindamycin, ciprofloxacin, gatifloxacin, levofloxacin, and tetracycline were determined following the CLSI recommendations (36). The breakpoints for fusidic acid, vancomycin, rifampin, and erythromycin were adopted from a previously published study (37, 38).

**TABLE 1:** Susceptibility-toxin genotype correlation in 16 clinical *C. difficile* isolates

Antibiotics	MIC breakpoint (μg/mL)^*a*^	No. (%) resistant				Analysis results	
		Total (*n* = 16)	A^−^B^+^ (*n* = 10)	A^+^B^+^ (*n* = 2)	A^−^B^−^ (*n* = 4)	*χ* ^2^	*P*
Metronidazole	S ≤ 8 R ≥ 32	0 (0.00)	0 (0.00)	0 (0.00)	0 (0.00)	N/A^*b*^	N/A
Vancomycin	S ≤ 2 R ≥ 32	0 (0.00)	0 (0.00)	0 (0.00)	0 (0.00)	N/A	N/A
Levofloxacin	S ≤ 2 R ≥ 8	1 (6.25)	0 (0.00)	1 (6.25)	0 (0.00)	4.709	0.095
Ciprofloxacin	S ≤ 2 R ≥ 8	14 (87.5)	9 (56.25)	2 (12.5)	3 (18.75)	1.626	0.444
Moxifloxacin	S ≤ 2 R ≥ 8	1 (6.25)	0 (0.00)	1 (6.25)	0 (0.00)	4.709	0.095
Gatifloxacin	S ≤ 2 R ≥ 8	1 (6.25)	0 (0.00)	1 (6.25)	0 (0.00)	4.709	0.095
Clindamycin	S ≤ 2 R ≥ 8	14 (87.5)	9 (56.25)	2 (12.5)	3 (18.75)	1.626	0.444
Erythromycin	S < 8 R ≥ 8	5 (31.25)	1 (6.25)	2 (12.5)	2 (12.5)	9.354	0.009
Fusidic acid	S ≤ 2 R ≥ 2	16 (100.00)	10 (62.50)	2 (12.5)	3 (18.75)	N/A	N/A
Rifampin	S ≤ 2 R ≥ 2	0 (0.00)	0 (0.00)	0 (0.00)	0 (0.00)	N/A	N/A
Tetracycline	S ≤ 4 R ≥ 16	2 (12.5)	0 (0.00)	2 (12.5)	0 (0.00)	12.057	0.002
Piperacillin	S ≤ 16 R ≥ 128	0 (0.00)	0 (0.00)	0 (0.00)	0 (0.00)	N/A	N/A

^*a*^ Resistance breakpoints were interpreted according to the corresponding references in Materials and Methods. S, susceptible; R, resistant.

^*b*^ N/A, not applicable.

### Statistical analysis

This study incorporates the screened positive bacterial strains into the analysis of CDC (9). Demographic and clinical characteristics of all patients were presented as medians with interquartile ranges (M [P25, P75]) for continuous variables and as frequencies with percentages (*n* [%]) for categorical variables. Descriptive statistics were employed to summarize demographic and clinical variables, presenting data as percentages, interquartile ranges, and frequencies. Univariate logistic regression analyses were first performed to identify risk factors associated with *C. difficile* carriage, with variables showing a *P* < 0.05 selected for inclusion in the final model. A multivariable logistic regression model was then constructed using a forward stepwise approach to examine the associations between independent variables and the outcome. The strength of these associations was quantified by calculating adjusted odds ratios (ORs) with corresponding 95% confidence intervals. In the multivariable analysis, variables with *P* < 0.05 were considered statistically significant.

Data were analyzed using the Statistical Package for Social Sciences (v23.0; SPSS, Armonk, NY, USA). After normality testing, univariate and multivariate logistic regression analyses were performed to assess the impact of different risk factors on CDC, and *P* < 0.05 was considered to indicate a statistically significant difference.

## RESULTS

### Enrollment of patients and collection of *C. difficile* isolates

**Fig 1:**
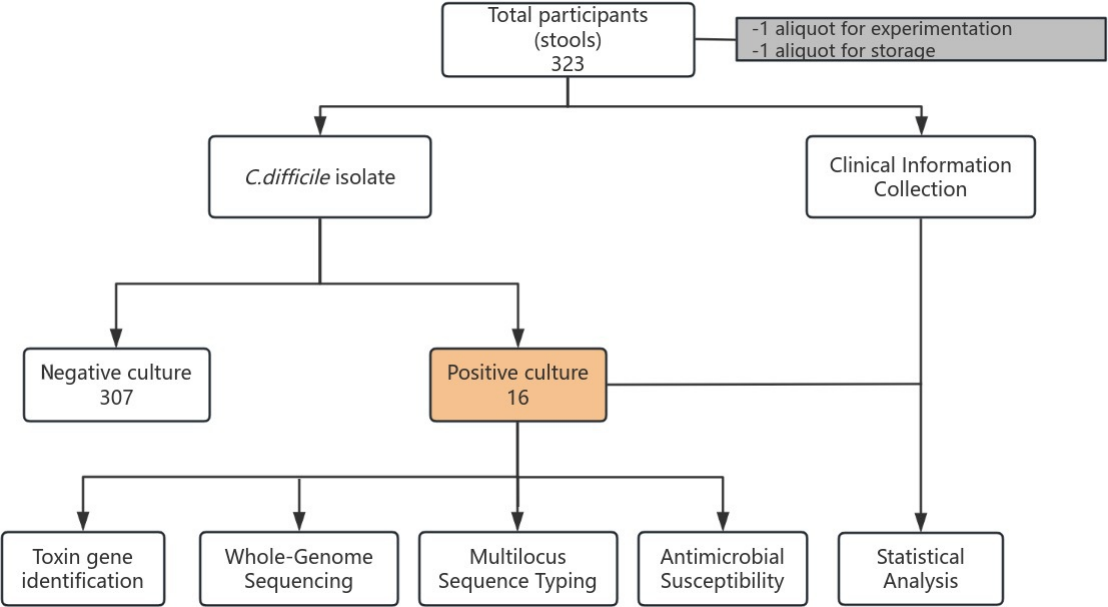
Flow diagram of data collected during this cross-sectional study.

A total of 323 patients diagnosed with chronic kidney disease were enrolled from the Nephrology Department of Anji County over 2 years (Fig. 1). The cohort comprised 235 cases of chronic nephritis, 84 cases of uremia, 2 cases of ANCA-associated glomerulonephritis, 1 case of Sjögren’s syndrome, and 1 case of post-renal transplantation. Among them, 78.95% (255/323) were male, and 75.85% (245/323) were aged 60 years or older.

### Toxin genes and MLST for *C. difficile* isolates

**Fig 2:**
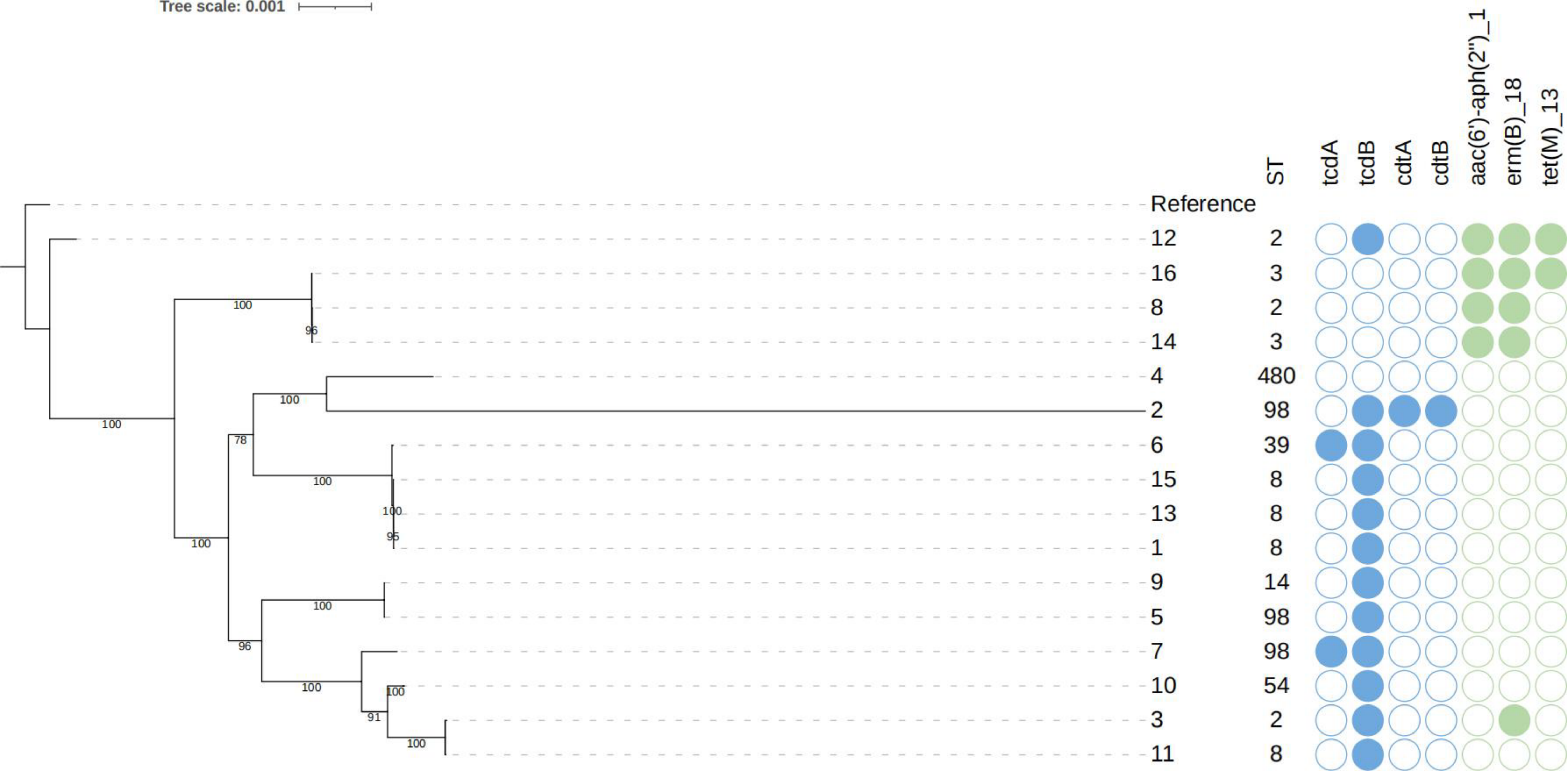
*In silico*-predicted distribution of antimicrobial resistance genes and virulence loci among the 16 *C. difficile* isolates. This heatmap illustrates the distribution of antimicrobial resistance genes and virulence loci in the whole-genome sequencing data. The presence of a gene is indicated by a blue bar, while the absence is indicated by a white bar.

Molecular typing of *C. difficile* isolates from patients with CKD revealed that MLST results classified the 16 strains into 8 distinct sequence types, indicating high genotypic diversity among the CDC strains. Among the 16 *C. difficile* isolates, 9 (56.25%) were A^−^B^+^CDT^−^, 1 (6.25%) was A^−^B^+^CDT^+^, 4 (25.0%) were A^−^B^−^CDT^−^, and 2 (12.5%) were A^+^B^+^CDT^−^ (Fig. 2).

### Phylogenomic and antimicrobial resistance gene analysis

Phylogenetic and genotypic analyses were performed on a total of 16 *C. difficile* genomes. As shown in Fig. 2, phylogenetic trees constructed based on whole-genome or core-genome single nucleotide polymorphisms revealed that all the genomes were divided into two major evolutionary clades. The reference strain formed a separate clade, while the remaining strains clustered into another clade with several subclusters within it (Fig. 2). Minimum spanning tree analysis demonstrated that none of the STs formed clonal complexes. Analysis of antimicrobial resistance genes, including *aac*(*6*′)*-aph*(*2*″) _1, *erm*(B) _18, and *tet*(M) _13, revealed that these strains generally did not carry multiple antibiotic resistance determinants. Strains 12 and 16 showed distinct distribution patterns for *aac*(*6*′)*-aph*(*2*″) _1, *erm*(B) _18, and *tet*(M) _13 (Fig. 2).

### Antimicrobial susceptibility of isolates

Antimicrobial susceptibility testing results (Table 1) showed that all isolates were 100% susceptible to metronidazole, vancomycin, piperacillin, and rifampin. However, universal resistance (100%, 16/16) was observed to fusidic acid, and high resistance rates (both 87.5%, 14/16) were detected against ciprofloxacin and clindamycin. In contrast, low resistance rates (all 6.25%, 1/16) were noted for levofloxacin, moxifloxacin, and gatifloxacin. Additionally, the resistance rates to erythromycin and tetracycline were 31.25% (5/16) and 12.5% (2/16), respectively. Notably, 31.25% of the isolates exhibited high-level resistance to clindamycin, with a MIC exceeding 128 µg/mL.

Further analysis of the association between antimicrobial resistance phenotypes and toxin genotypes (A^−^B^+^, A^+^B^+^, and A^−^B^−^) revealed that the distribution of resistance to erythromycin and tetracycline exhibited statistically significant differences among different genotype groups (*P* < 0.05). In contrast, no significant differences were observed in the resistance rates to ciprofloxacin, clindamycin, and moxifloxacin across the various toxin genotype groups (*P* > 0.05) (Table 1).

### Assessment of risk factors

Univariate analysis of the 323 patients identified several demographic and clinical factors associated with CDC (Table 2), including Sjögren’s syndrome (OR = 1.067; 95% CI, 0.940–1.211), hyperphosphatemia (OR = 4.901; 95% CI, 1.320–12.408), renal function stage (*P* = 0.027), and dialysis frequency (*P* = 0.038). Sjögren’s syndrome, hyperphosphatemia, and dialysis frequency were included in the multivariable logistic regression model (renal function stage was excluded due to concerns of multicollinearity) (Table 3). Hyperphosphatemia was associated with a statistically significant increased risk of CDC (OR = 1.178; 95% CI, 1.048–1.065). A hemodialysis frequency of four times per day also significantly increased the risk of CDC (OR = 1.104; 95% CI, 1.023–1.467).

**TABLE 2:** Characteristics and risk factors of *C. difficile* colonization in 323 CKD patients

Factor^*a*^	*C. difficile* positive (*n* = 16)	*C. difficile* negative (*n* = 307)	*χ* ^2^	OR	95% CI	*P* value
Age (IQR: 60–77)			0.343			0.511
<60	2 (12.5)	70 (23.8)				
≥60	14 (87.5)	231 (76.2)				
Sex (male)	12 (75.0)	243 (79.2)	0.007	0.83	0.35–2.00	0.934
Fever	3 (18.8)	36 (11.7)	0.200	1.74	0.47–6.39	0.655
Antibiotic use (<90 days)	1 (6.3)	13 (9.7)	0.00	1.51	0.19–12.30	1.000
Carbapenems	0 (0.00)	1 (10.00)				
Oxazolidinones	0 (0.00)	1 (10.00)				
Nitroimidazoles	0 (0.00)	1 (10.00)				
Penicillins	0 (0.00)	2 (20.00)				
Cephalosporins	0 (0.00)	2 (20.00)				
Fluoroquinolones	0 (0.00)	2 (20.00)				
β-Lactamase inhibitors	1 (100.00)	1 (10.00)				
Surgical operation (>12 months)	1 (0.07)	2 (0.01)	0.882	10.17	0.87–118.50	0.348
Dialysis			0.092	0.68	0.19–2.44	0.742
Yes	3 (18.8)	78 (25.4)				
No	13 (81.3)	229 (74.6)				
Dialysis duration (>12 months)	2 (3.9)	49 (96.1)	0.843			0.656
Dialysis frequency			21.442			0.000
4 times/day	3 (0.21)	6 (0.02)				
3 times/week	0 (0.00)	70 (0.23)				
Disease diagnosis						
Chronic nephritis	12 (75.0)	223 (72.6)	0.00	1.13	0.36–3.60	1.000
Uremia	3 (18.8)	81 (26.4)	0.149	0.64	0.18–2.32	0.699
ANCA-associated glomerulonephritis	0 (0.00)	2 (0.7)	0.00	0.99	0.99–1.00	1.000
Sjögren’s syndrome	1 (6.3)	0 (0.00)	4.323	1.07	0.94–1.21	0.038
Renal transplantation status	0 (0.00)	1 (0.3)	0.00	0.10	0.99–1.00	1.000
CKD staging			10.967			0.027
G1	6 (37.5)	35 (11.4)				
G2	3 (18.8)	52 (16.9)				
G3	3 (18.8)	86 (28.0)				
G4	0 (0.00)	48 (15.6)				
G5	4 (25.8)	86 (28.0)				
Laboratory results						
WBC (×10^9^ /L)	6.95 (4.70,9.98)	6 (4.80,8.40)	0.00	1.01	0.31–3.24	1.000
NEUT%	68.75 (63.07,76.15)	68.2 (59.70,68.20)	0.00	1.06	0.36–3.14	1.000
LYM%	22.9 (16.73,26.48)	21.6 (15,29.30)	0.906	0.60	0.20–1.75	0.341
Serum albumin	38.2 (36.38,43.83)	38.4 (34.10,42.10)	0.361	0.57	0.16–2.03	0.548
Serum creatinine	127.5 (94.75,491)	143 (88,339)	0.00	0.88	0.30–2.62	1.000
Serum potassium	3.91 (3.57,4.19)	4.06 (3.65,4.50)	0.626	0.42	0.10–1.99	0.429
FBG	6.415 (4.86,7.58)	5.850 (5.05,7.40)	1.243	1.79	0.64–5.05	0.265
Other diseases						
Nephropathy	14 (87.5)	231 (75.2)	0.668	2.30	0.51–10.36	0.414
Pneumonia	4 (25)	120 (39.1)	0.75	0.52	0.16–1.65	0.386
Liver disease	1 (6.3)	55 (17.9)	0.745	0.31	0.04–2.36	0.388
Diabetes mellitus	5 (31.3)	78 (25.4)	0.052	1.33	0.45–3.96	0.820
Hypertension	13 (81.3)	207 (67.4)	1.338	2.09	0.58–7.51	0.247
Heart disease	2 (12.5)	93 (30.3)	1.541	0.33	0.07–1.48	0.214
Hyperphosphatemia	5 (31.3)	31 (10.1)	4.901	4.05	1.32–12.40	0.027
Gastroenteritis (>12 months)	2 (12.5)	41 (13.4)	0.00	0.93	0.20–4.23	1.000

^*a*^ WBC, white blood cell; NEUT%, neutrophil ratio (%); LYM%, lymphocyte ratio (%); and FBG, fasting blood glucose.

**TABLE 3:** Binary multivariate logistic regression analysis

Variable	*B*	S.E	Wald *χ*²	*P*	Exp(*B*)	95% CI	
						Lower limit	Upper limit
Sjögren’s syndrome	−24.378	40,192.934	0.00	1.00	0.00	0.00	0.00
Dialysis frequency	2.264	0.767	8.725	0.003	1.104	1.023	1.467
Hyperphosphatemia	1.727	0.668	6.687	0.010	1.178	1.048	1.065

## DISCUSSION

This study characterized the carriage rate, antimicrobial resistance profile, and associated risk factors of CDC among CKD outpatients in eastern China. The CDC carriage rate in our study was lower than the rates reported in previous studies on CKD patients—particularly high-risk subgroups such as those with end-stage renal disease or kidney transplant recipients (39–41). These higher rates are likely associated with dialysis-associated intestinal mucosal ischemia and transplant-related immunosuppression, which can disrupt the gut barrier. In contrast, we identified a lower positive rate of 5.0% in our specific outpatient cohort. The discrepancy may reflect population heterogeneity (42). Unlike previous studies focused on high-risk hospitalized, dialysis, or post-transplant patients, our cohort primarily consisted of clinically stable outpatients. Our findings suggest that the burden of CDC may be correspondingly lower in renal patients with less advanced disease and minimal immunosuppressive exposure. These observations underscore the importance of distinguishing between CKD stages, treatment modalities, and underlying immune status for an accurate assessment of CDC epidemiology (43, 44).

In this study, MLST and antimicrobial susceptibility testing were performed on 16 clinical isolates of *C. difficile*. Molecular typing results revealed that all isolates were predominantly clustered within MLST clade 1, indicating that this clade represents the major epidemic lineage in the local region. In China, ST3, ST35, and ST54 are the dominant strains, all of which belong to clade 1 (45), and the resistance profiles of the isolates in this study are generally similar to those reported in previous studies in China (17, 45). Although all isolates remained susceptible to first-line agents (metronidazole and vancomycin), the clade 1 lineage still poses a potential transmission risk due to a certain CDI recurrence rate (45–47). Among these isolates, the distribution of STs was scattered rather than highly concentrated. In addition, it is noteworthy that these isolates shared similar molecular characteristics with ST2 characterized in our previous study conducted in China, exhibiting low genetic diversity in the mutations associated with antibiotic resistance (48, 49). Notably, this study observed higher resistance rates to fusidic acid (100% compared with 51.5%), ciprofloxacin (87.5% compared with 78.8%), and clindamycin (87.5% compared with 63.6%) than our previous study involving colorectal cancer patients (49). CKD and cancer patients belong to immunocompromised populations with higher antibiotic exposure and more frequent hospitalizations (18, 42). Given that patients with CKD generally have longer survival than those with cancer, it is reasonable that patients with CKD have a longer time window for antibiotic exposure and hospital admissions. Furthermore, no association was found between antibiotic resistance profiles of *C. difficile* isolates and prior antibiotic exposure in the corresponding patients. This rising trend suggests that within the healthcare environment under investigation, there may be selective pressure driving the development of resistance to these antimicrobial agents, warranting continued surveillance (18, 45, 50).

Antimicrobial susceptibility testing showed that all isolates remained fully susceptible to first-line agents (metronidazole and vancomycin). However, universal resistance was observed to fusidic acid, alongside high resistance rates to ciprofloxacin and clindamycin (87.5% each), with 31.3% of isolates exhibiting high-level clindamycin resistance. This profile resembles that of globally circulating hypervirulent *C. difficile* strains, suggesting similar resistance traits in local clones, likely driven by prior antimicrobial selection (51, 52). Notably, resistance to erythromycin and tetracycline varies significantly by toxin genotype. Both tetracycline-resistant isolates clustered in the A^+^B^+^ group, implying a possible genetic link between this toxin type and specific resistance. A similar study found that among 67 toxigenic *C. difficile* isolates, 6 strains with the A^+^B^+^CDT^−^ genotype belonging to ST3 exhibited reduced susceptibility to tetracycline (MIC = 8 mg/L) (53), supporting our finding that tetracycline resistance may not be randomly distributed across different toxin genotypes, which should be further verified. In contrast, ciprofloxacin and clindamycin resistance was widespread across all genotypes, indicating these are now common features of local strains. Moreover, the *C. difficile* carriage rate in healthy Chinese populations is approximately 0.7%–5.5%, and *C. difficile* isolates from healthy individuals exhibit substantially lower resistance rates (62.55%) to fluoroquinolones and clindamycin than that observed (87.5%) in our CKD outpatient cohort (16, 54, 55). Thus, our findings reveal a dual pattern of retained first-line susceptibility alongside established multidrug resistance. Although the association between the A^+^B^+^ genotype and tetracycline resistance may serve as a valuable marker for tracking resistance spread and has potential implications for local treatment and infection control, this finding should be interpreted with caution owing to the small number of positive isolates, and further validation in larger cohorts should be required (12, 56–59).

For risk factor analysis, hyperphosphatemia, advanced chronic kidney disease, and dialysis frequency were identified as factors significantly associated with *C. difficile* carriage. This aligns with the established consensus that CKD patients represent a high-risk population for CDI (10, 60–63), although this conclusion is limited by the study population. Multivariable analysis confirmed hyperphosphatemia and high-frequency hemodialysis as independent risk factors. To our knowledge, this study is the first to identify hyperphosphatemia and high-frequency hemodialysis as independent risk factors for CDC in CKD outpatients. Hyperphosphatemia, a key marker of CKD progression and metabolic dysregulation, reflects a poorer overall physiological status (64), in which *C. difficile* spore germination is promoted by altering intestinal pH and disrupting the gut epithelial barrier (65, 66). Meanwhile, high-frequency dialysis likely serves as a proxy for both disease severity and increased healthcare exposure. Previous studies have demonstrated that patients undergoing more frequent dialysis have prolonged contact with hospital environments, where contamination with *C. difficile* spores represents a well-documented route of acquisition (67, 68). Given the relatively small sample size of our colonized cohort, these preliminary findings should be interpreted with caution and warrant validation in larger, prospective studies.

This study provides the first description of molecular epidemiology for CDC in a chronic nephritis population within a primary care setting in eastern China. Hyperphosphatemia and high-frequency hemodialysis were independent risk factors for CDC. The specific association between the toxin genotype and tetracycline resistance, alongside the high-risk clinical profile characterized by frequent dialysis and hyperphosphatemia, provides actionable targets for future interventions. Although this study has limitations, including a single-center, cross-sectional design and a relatively small sample size of CDC-positive isolates, the findings provide crucial local baseline data for infection control in community hospitals and highlight the need for multicenter prospective studies to validate the results. In addition, these preliminary observations also indicate that active surveillance and risk-stratified strategies should be conducted in other immunocompromised populations.

## Data Availability

Due to the sensitive nature of the data (e.g., patient information), access is restricted. Data are available from the corresponding author upon reasonable request and subject to approval by the Ethics Committee of the Third People's Hospital of Anji County.
